# The genetic epidemiology of schizotypal personality disorder

**DOI:** 10.1017/S0033291724000230

**Published:** 2024-07

**Authors:** Kenneth S. Kendler, Henrik Ohlsson, Jan Sundquist, Kristina Sundquist

**Affiliations:** 1 Department of Psychiatry, Virginia Institute for Psychiatric and Behavioral Genetics, Virginia Commonwealth University, Richmond, VA, USA; 2 Department of Psychiatry, Virginia Commonwealth University, Richmond, VA, USA; 3 Center for Primary Health Care Research, Lund University, Malmö, Sweden; 4 University Clinic Primary Care Skane, Sweden; 5 Department of Population Health Science and Policy, Icahn School of Medicine at Mount Sinai, NY, USA

**Keywords:** conversion to schizophrenia, comorbidity, genetic risk, Schizotypal personality disorder, Sweden

## Abstract

**Background:**

The concept of schizotypal personality disorder (SPD) emerged from observations of personality characteristics common in relatives of schizophrenic patients. While often studied in family designs, few studies and none with genetic measures, have examined SPD in epidemiological samples.

**Methods:**

We studied individuals born in Sweden 1940–2000 with an ICD-10 diagnosis of SPD with no prior schizophrenia (SZ) diagnosis (*n* = 2292). Demographic features, patterns of comorbidity, and Family Genetic Risk Scores (FGRS) were assessed from multiple Swedish registries. Prediction of progression to SZ was assessed by Cox models.

**Results:**

SPD was rare, with a prevalence of 0.044%, and had high levels of comorbidity with autism spectrum disorder (ASD), OCD, ADHD, and major depression (MD), and increased rates of being single, unemployed and in receipt of welfare. Affected individuals had elevated levels of FGRS for SZ (+0.42), ASD (+0.30), MD (+0.29), and ADHD (+0.20). Compared to cases of schizophrenia, they had significantly lower rates of FGRS_SZ_, but significantly elevated rates of genetic risk for ASD, MD, and ADHD. Over a mean follow-up of 8.7 years, 14.6% of SPD cases received a first diagnosis of SZ, the risk for which was significantly increased by levels of FGRS_SZ_, male sex, young age at SPD diagnosis and an in-patient SPD diagnosis and significantly decreased by comorbidity with MD, ASD, and ADHD.

**Conclusions:**

Our results not only support the designation of SPD as a schizophrenia spectrum disorder but also suggest potentially important etiologic links between SPD and ASD and, to a lesser extent, ADHD, OCD, and MD.

*In many instances it has been observed that, while some members of a family are manifest lunatics, others are only eccentric in character, displaying peculiarities of habit, conduct, and disposition*. (Prichard, 1837) p. 124.

… *Every psychiatrist is aware how frequently especially the relatives of dementia praecox patients present deviations from normal mental behavior, which can be described as ‘abnormal characteristics’, but who in no way are to be viewed as fully schizophrenic*. (Hoffmann, 1921) p.7; (Kendler and Klee, 2022b)

*… the studies on empirical hereditary prognosis have shown that in the sphere of relatives of schizophrenics, this type of* [*schizoid/schizotypal*] *psychopath has a special position in terms of genetics. If we were to describe people who we do not know as blood relatives of schizophrenics as ‘schizoid’ psychopaths, we would say something about them that we cannot prove, namely their genetic relationship to schizophrenics. Such people, no matter how clearly they display a ‘schizoid’ nature, should not be assigned this genetically prejudicial name, but rather classify them purely descriptively typologically.* (Luxenburger, 1939) (translation by A Klee and KSK) p. 812-3

The origins of our current concept of Schizotypal Personality Disorder (SPD), as outlined in DSM-5 (American Psychiatric Association, 2013) and ICD-10 (Organization, 1992), can be traced to many comments from psychiatrists in the 19th century who, like Prichard, noted an excess of unusual character traits in relatives of their ‘insane’ asylum patients followed by systematic observations in the first set of family studies employing dementia praecox probands conducted in Munich by a number of investigators, including Hoffmann, in the first decades of the 20th century.

The familial/genetic link to schizophrenia was also employed in the development of the DSM-III criteria for SPD, the forerunner of the current DSM-5 and ICD-criteria. These criteria were based on personality traits observed in the cases of ‘borderline schizophrenia’ in the Danish Adoption study of schizophrenia (Spitzer, Endicott, & Gibbon, 1979) that had been shown to aggregate strongly in the biological relatives of the schizophrenia adoptees (Kety, Rosenthal, Wender, & Schulsinger, 1971). Thus, the SPD diagnosis was created to capture the clinical features of individuals with a strong genetic risk for schizophrenia (Kendler, 1985). Many studies have since shown that SPD does indeed substantially aggregate in the close relatives of individuals with schizophrenia (Asarnow et al., 2001; Baron, Gruen, Asnis, & Kane, 1983; Kendler, Gruenberg, & Strauss, 1981; Kendler et al., 1993).

However, as pointed out by the above 1939 quote from Luxenburger – best known for conducting the first-ever systematic twin study of SZ (Luxenburger, 1928) (Kendler and Klee, 2022a) — it is one thing to show that SPD is increased in relatives of schizophrenia probands and another to show that SPD, ascertained from a population-based sample, bears a substantial genetic risk for schizophrenia. There might, for example, be multiple developmental pathways to SPD in the general population many of which do not depend on an elevated genetic risk for schizophrenia. Support for this hypothesis comes from the weak and/or inconsistent evidence that individuals selected solely for scoring high on self-report schizotypy scales have elevated schizophrenia polygenic risk scores (Docherty et al., 2020; Hatzimanolis et al., 2018; Mas-Bermejo et al., 2023) and epidemiological evidence that schizotypal/psychotic-like symptoms may not be at all specific to a liability to schizophrenia but rather may lie on the continuum of neuroticism and precede or follow the onset of a range of nonpsychotic psychiatric disorders (McGorry, Hartmann, Spooner, & Nelson, 2018; McGrath et al., 2016; Saha, Scott, Varghese, & McGrath, 2011; Zoghbi et al., 2019).

This report takes a different and to our knowledge novel approach to the study of SPD – to examine it in a general population sample (which has been done at least three times previously (Hastrup, Jennum, Ibsen, Kjellberg, & Simonsen, 2021; Hjorthøj, Albert, & Nordentoft, 2018; Köhler-Forsberg et al., 2023)) with available genetic risk measures. We ask the following major questions:

1. For SPD, what is its prevalence, age at onset, patterns of comorbidity (examining specifically those disorders previously suggested to be associated with SPD: autism spectrum disorder (ASD) (Barneveld et al., 2011; Esterberg, Trotman, Brasfield, Compton, & Walker, 2008), ADHD (Bernardi et al., 2012; Fagel et al., 2013), OCD (Attademo & Bernardini, 2021), and major depression (MD) (Rosell, Futterman, McMaster, & Siever, 2014)), and levels of social functioning?
2. In cases of SPD, what is their average genetic risk of schizophrenia and the potentially comorbid disorders of ASD, ADHD, OCD, and MD, and how does this profile of genetic risk compare with that of a matched sample of patients with schizophrenia?
3. What proportion of cases of SPD progress to a diagnosis of schizophrenia and to what degree can demographic, clinical, and levels of genetic risk predict this progression? More specifically can we replicate prior evidence that among cases of SPD, drug use disorder predicts the development of schizophrenia?(Hjorthøj et al., 2018)

## Methods

**Figure 1.**
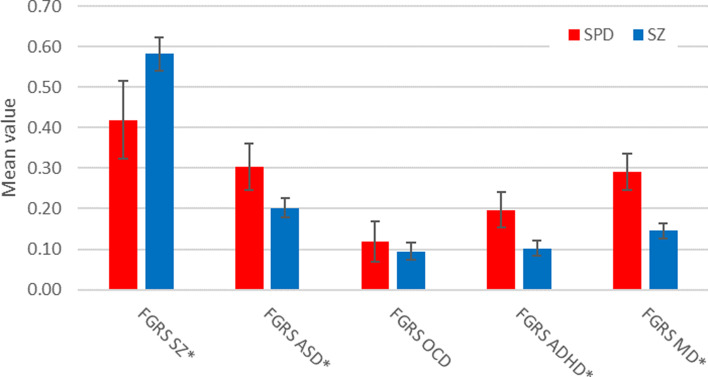
Mean (and 95% CIs) for the familial genetic risk score, depicted on the Y-axis, for schizophrenia (SZ), autism spectrum disorder (ASD), obsessive-compulsive disorder (OCD), ADHD, and major depression (MD) and in cases of schizotypal personality disorder (SPD) and schizophrenia in the Swedish population born 1940–2000 by ICD-10 (for exact values of these results see online Supplementary appendix table 5). * FRGS scores for SPD and schizophrenia differ significantly.

We collected information on individuals from Swedish population-based registers with national coverage linking each person's unique personal identification number which, to preserve confidentiality, was replaced with a serial number by Statistics Sweden (online Supplementary appendix table 1 and figure 1). We secured ethical approval for this study from the Regional Ethical Review Board in Lund and no participant consent was required (No. 2008/409 and later amendments). Our database consisted of all individuals born in Sweden between 1940 and 2000, to Swedish born parents. In the database, we included registrations for Schizotypal Personality Disorder (SPD) and schizophrenia (SZ). SPD was only available in ICD 10 (1997 to 2018), while for SZ we could also use ICD8 and ICD9 (registrations going back to 1973). See online Supplementary appendix table 2 for definitions of these variables.

For our first question (demographic features of SPD cases), we considered individuals with SPD without a prior registration of SZ. To compare these individuals with the general population we matched the cases of SPD with five individuals without a registration for SPD with the same year of birth, same sex, and same municipality of residence at the time of the cases' SPD registration. For SPD cases and their controls, we included lifetime registrations for MD, OCD, ADHD, and ASD in the database. Furthermore, we included information on social welfare, unemployment status, marital status, educational attainment, and grades from lower secondary school (at age 16). We used conditional logistic regression to calculate Odds Ratios (OR) by comparing SPD cases and their controls.

For the second question (genetic risk profiles), we created a database including the SPD cases and individuals registered for SZ during the same period (1997 to 2018). In the database, we included individual family genetic risk scores (FGRS) for SZ, MD, ASD, OCD, and ADHD, respectively. The FGRS is an estimate of an individual's genetic liability that reflects the relative lack or excess of disease in their pedigree relative to population expectations. The FGRSs are calculated from morbidity risks in first- through fifth-degree relatives, controlling for cohabitation effects, and thus arise from phenotypes in extended pedigrees (see online Supplementary Appendix table 3), not from molecular genetic data. We calculated the least squares means (95% CIs) for all FGRSs for the SPD and SZ cases while controlling for year of birth and sex. To evaluate potential genetic heterogeneity among SPD cases, we performed a Latent class analysis using the 4 FGRSs (OCD, ASD, ADHD, and SZ) as observed variables. We categorized the FGRSs into 4 classes based on k-means clustering before entering them into the LCA (see online Supplementary appendix table 4). The number of latent classes was determined by comparing model fit statistics between nested models. Improvement in model fit is indicated by smaller values of, Akaike's Information Criterion (AIC) (Akaike, 1987), Bayesian information criterion (BIC) (Vrieze, 2012), and entropy values close to 1.0.

For the third major question (conversion to SZ among SPD cases), we selected SPD cases born between 1960 and 2000 because earlier cohorts had a truncated follow-up given late ages at first registration due to the availability of ICD-10 only in 1997. We used a Cox regression model to investigate time to SZ where we followed individuals to date of SZ, date of death, date of emigration, or end of follow-up (2018-12-31) whichever came first. We performed a series of univariable models with the demographic features, the FGRS profiles, educational variables, the comorbidity variables, and the site of diagnosis (e.g. inpatient, primary care) of SPD as covariates. All statistical analyses were performed using SAS 9.4 (SAS Institute, 2012).

## Results

### Demographic features

We found in our cohort 2952 individuals diagnosed with SPD, 660 (22.3%) of whom had a prior diagnosis of SZ and therefore were not further considered. Using the remaining cases (*n* = 2292; 47.5% male), SPD had a population prevalence of 0.044%. The site of registration for SPD, using a hierarchy, was inpatient *n* = 531 (23.2%), specialist (including psychiatrists) *n* = 784 (34.2%), and primary care *n* = 977 (42.6%). Estimation of an accurate age at first diagnosis was problematic given that diagnoses were only available from 1997–2018. This is likely best captured by our 1980–89 cohort where the mean (s.d.) equaled 25.8 (4.4) years.

We examined the rates of four psychiatric disorders suspected of being comorbid with SPD in our SPD cohort compared to matched controls (Table 1). All were substantially more common in our SPD with the ORs being strongest for ASD, followed by OCD, ADHD, and finally MD. Compared to controls, individuals with SPD were significantly more likely to receive social welfare, to be single, to be divorced if married, to have low academic performance at age 16, and to have low educational attainment. They also had moderately elevated rates of unemployment.

**Table 1.** Comparison with the general population (SPD individuals matched to five controls (same year of birth, same sex, and same municipality (at time of SPD registration)

	SPD (%) population	Matched sample (%)	Odds ratio (95% CI)
Major depression	59.8	17.7	7.45 (6.70; 8.27)
OCD	9.9	0.9	11.6 (9.13; 14.8)
ADHD	16.4	2.1	10.0 (8.33; 12.0)
Autism spectrum disorder	13.8	0.8	20.4 (15.9; 26.3)
Not married	71.0	44.0	3.94 (3.53; 4.39)
Divorced (only married individuals)	63.3	30.6	4.54 (3.68; 5.61)
Social welfare	55.3	15.2	8.21 (7.35; 9.18)
Unemployment	57.0	43.9	1.83 (1.66; 2.02)
Low education*	27.8	10.8	3.38 (3.01; 3.79)
Low grades*	12.8	5.9	2.69 (2.29; 3.16)

*Low grades and low education are defined as more than 1 SD below the mean score.

### Genetic risk profile for SPD

We present in Fig. 1, the mean (± 95% CIs) FGRS for our cases of SPD for schizophrenia, ASD, OCD, ADHD, and MD. The rates were substantially and significantly elevated for schizophrenia followed by ASD and MD. Elevations were more modest for ADHD and especially OCD. We also examined the mean (±95% CIs) levels of the FRGS_SZ_ as a function of the site of SPD diagnosis. No significant difference was observed across the three groups: in-patient 0.41 (0.24–0.59); specialist 0.44 (0.29–0.59) and primary care 0.43 (0.30–0.56).

### A comparison of the genetic risk profile for SPD and schizophrenia

We then calculated the FGRS for the same set of four disorders in cases of schizophrenia ascertained over the same registration period (*n* = 12 466) and compared them to those found for SPD (Fig. 1). The genetic risk for schizophrenia was substantially and significantly higher in the schizophrenic *v.* the SPD cases while the reverse pattern was seen for the genetic risk for ASD, ADHD, and MD. The genetic risk for OCD did not differ in the two proband groups.

### Evaluation of possible clinician bias in SPD diagnosis

Because the FGRS is based on recorded diagnoses in the extended family and not molecular genetic data, it is possible that Swedish clinicians were more likely to diagnose SPD in individuals with a close relative with schizophrenia, thereby biasing upward their apparent genetic risk. To evaluate this hypothesis, we recalculated the FGRS_SZ_ in our samples for SPD and, as a comparison, for schizophrenia after stripping out information on all first-degree relatives. The mean (±95% CIs) FGRS in our SPD cases declined very modestly from 0.42 (0.32–0.52) to 0.39 (0.29–0.48)], with a similar small decline seen in our schizophrenia cases, from 0.58 (0.54–0.62) to 0.54 (0.50–0.58).

### Genetic heterogeneity in SPD

Given that our SPD cohort has elevated genetic risk for four relatively uncommon disorders (SZ, ASD, OCD, and ADHD), we sought to evaluate the hypothesis that our sample is genetically heterogeneous and can be cleanly divided into more homogeneous groups each with a substantial elevation of genetic risk for only one of these disorders. We therefore performed a latent class analysis (LCA) of these four FGRSs each divided into four levels based on K-means clustering. By all three of our fit indices (AIC, BIC, and entropy), the three class solution fitted best (online Supplementary Appendix table 6). For simplicity of interpretation, we focus on the combination of the medium high and high groups as seen in Table 2, our three classes were easily named as those at (i) generally low genetic risk (67% of the sample), (ii) intermediate genetic risk (26% of the sample), and (iii) high genetic risk (7% of the sample). The low-risk group contained only a small proportion of individuals (all <4.0%) with substantially elevated genetic risks for our four disorders. In the intermediate-risk group, 5–21% of the sample had substantially elevated genetic risk to one of the four disorders, with those at high genetic risk for ADHD and ASD being the most common. In those at high genetic risk, 17–3% of the subjects were at particularly high genetic risk for one of our four disorders, with high risk being most common for ASD and SZ.

**Table 2.** Results from three-class solution of the latent class analysis of cases of SPD

Class name	Low genetic risk	High genetic risk	Intermediate genetic risk
Group Percentage	66.9%	6.7%	26.4%
	Item response probabilities		
Low FGRS			
OCD	93.2%	82.8%	74.5%
ASD	97.8%	44.3%	34.5%
ADHD	82.7%	57.4%	33.2%
SZ	92.6%	70.9%	82.1%
Mid Low FGRS			
OCD	5.3%	0.1%	19.3%
ASD	2.2%	3.2%	50.9%
ADHD	13.6%	1.7%	46.2%
SZ	4.8%	0.5%	12.6%
Mid High FGRS			
OCD	1.2%	12.7%	6.0%
ASD	0.0%	36.9%	14.0%
ADHD	3.5%	26.9%	17.6%
SZ	1.9%	18.9%	5.4%
High FGRS			
OCD	0.2%	4.3%	0.3%
ASD	0.0%	15.6%	0.6%
ADHD	0.2%	0.1%	3.0%
SZ	0.7%	9.6%	0.0%
Sum of Mid-High and High FGRS			
OCD	1.4%	17.0%	6.3%
ASD	0.0	52.5%	14.6%
ADHD	3.7%	27.0%	20.6%
SZ	2.6%	28.5%	5.4%

### Progression to schizophrenia over time

**Figure 2.**
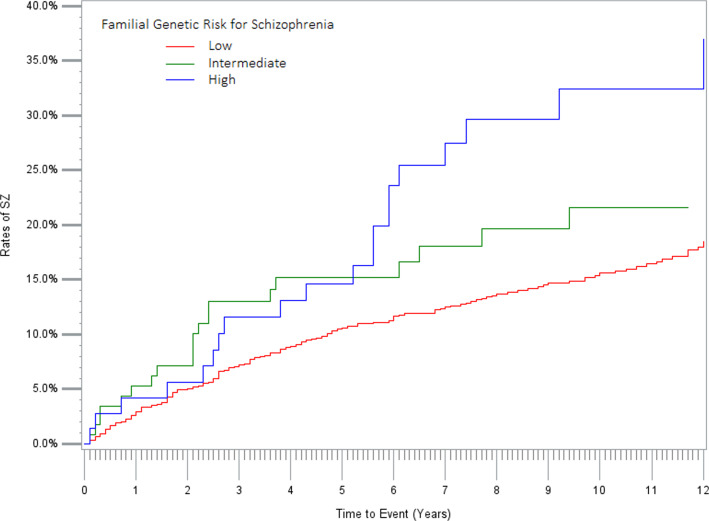
Survival curves for progression to a diagnosis of schizophrenia In cases of SPD with low, intermediate, and high genetic risk for Schizophrenia as calculated by a Cox model. The X-axis represents years since first diagnosis and the Y axis the Proportion who received a first diagnosis of schizophrenia.

For these analyses, we examined all SPD cases born in 1960 and onwards (*n* = 1664). By the end of our follow-up period, a mean (s.d.) of 8.7 (5.3) years, 243 individuals (14.6%) had received a first diagnosis of SZ. Table 3 presents a univariable Cox regression analysis predicting the conversion of SPD to SZ in our sample. Progression was significantly predicted by all three demographic variables: earlier year of birth, younger age at first SPD diagnosis, and, particularly strongly, male sex. Of the five genetic risk factors examined, now including drug use disorder, only risk for schizophrenia significantly predicted progression of SPD to schizophrenia. We illustrate the impact of this effect in Fig. 2, with survival curves for three groups of the SPD cases at low, moderate, and high FGRS_SZ_. Twelve years after the first SPD diagnosis, the risk for schizophrenia (±95%CI) in the three groups were, respectively, 18.5% (95%CI: 16.0–21.4), 21.6% (14.2–32.1), and 36.9% (24.5–53.1). Poor academic performance at age 16 significantly protected against the development of schizophrenia. Compared to a diagnosis of SPD received in primary care, the rate of schizophrenia was substantially higher in those diagnosed in specialty and in-patient care. Comorbidity with MD, ADHD, and ASD but not OCD significantly reduced the probability of the progression to schizophrenia.

**Table 3.** Prediction of conversion of Schizotypal personality disorder to Schizophrenia

	Hazard Ratio for a First Diagnosis of Schizophrenia
Demographics	
Year of birth	0.97 (0.96; 0.99)
Age at SPD registration	0.91 (0.89; 0.94)
Male sex	1.57 (1.22; 2.03)
Genetic risk	
FGRS_SZ_	1.07 (1.02; 1.12)
FGRS_MD_	0.96 (0.84; 1.09)
FGRS_ASD_	0.98 (0.91; 1.07)
FGRS_OCD_	0.93 (0.83; 1.04)
FGRS_ADHD_	0.89 (0.79; 1.00^1^)
Educational variables	
Low grades (age 16) (*N* = 1000)	0.79 (0.66; 0.93)
Low parental education	0.87 (0.75; 1.01)
Type of registration	
-Inpatient	3.71 (2.62; 5.26)
-Specialized	2.01 (1.41; 2.87)
-Primary care	Ref
Comorbidity with	
MD	0.49 (0.38; 0.63)
ADHD	0.41 (0.27; 0.62)
ASD	0.68 (0.46; 0.99)
OCD	1.02 (0.69; 1.51)
DUD	1.19 (0.92; 1.53)

1 Upper CI limit = 1.001.

## Discussion

Using the Swedish medical and genealogical registries, we examined the genetic epidemiology of SPD as diagnosed by ICD-10 criteria. Of the many findings reported here, eight are noteworthy and are discussed in turn. First, SPD, as diagnosed in a clinical setting by a physician, was a quite rare disorder, diagnosed in around 0.04% of the Swedish population with a nearly equal prevalence in males and females. These estimates are much lower than the prevalence estimates of 1.6–3.9% obtained at personal interviews in four general population surveys in the US (Sansone & Sansone, 2011) and 0.6% found in one such survey in Norway (Torgersen, Kringlen, & Cramer, 2001).

Second, consistent with prior reports (Attademo & Bernardini, 2021; Barneveld et al., 2011; Esterberg et al., 2008; Rosell et al., 2014), we found that cases of SPD had, as a group, high levels of psychiatric comorbidity that were particularly prominent for ASD, OCD, ADHD, and MD.

Third, consistent with a prior Danish study (Hastrup et al., 2021), we found that SPD patients were relatively impaired, with poor marital, occupational, and educational functioning, and an over 8-fold elevation in receipt of social welfare.

Fourth, as diagnosed by Swedish clinicians, individuals with SPD are at a substantially elevated genetic risk for schizophrenia. This provides robust support, from an epidemiological sample, for the repeated observations in adoption and family studies for the close familial/genetic link between SPD and schizophrenia (Asarnow et al., 2001; Baron et al., 1983; Kendler et al., 1981; Kendler et al., 1993). The possibility that our schizophrenia genetic risk estimate for the SPD cases was upwardly biased by clinicians preferentially assigning an SPD diagnosis to cases with schizophrenic first-degree relatives was not supported by our analyses. Our robust evidence for a genetic link between SPD and schizophrenia stands in contrast to the weak indications of elevated schizophrenia polygenic risk scores (PRS) in population samples scoring high on self-report schizotypy scales (Docherty et al., 2020; Hatzimanolis et al., 2018; Mas-Bermejo et al., 2023). These findings might arise because relatives of schizophrenics are much better discriminated against from relatives of controls by the signs of schizotypy found at personal interviews, such as odd communication, poor rapport, guardedness, and oddness than by schizotypal symptoms assessed by questionnaire, such as magical thinking, ideas of reference and illusions (Kendler, McGuire, Gruenberg, and Walsh, 1995; Kendler, Thacker, and Walsh, 1996). This is, in turn, consistent with evidence for the lack of specificity of the polygenic risk from a large genetic association study of psychotic experiences in the UK Biobank (Legge et al., 2019). This study found support for a shared genetic liability between psychotic experiences and schizophrenia, major depressive disorder, bipolar disorder, ASD, and ADHD suggesting that SZ has a more specific genetic relationship with SPD than with the phenotype of psychotic experiences. Of note, the level of genetic risk in our SPD cases did not differ for those diagnosed in primary, specialist, or in-patient sites, suggesting that, at least from a genetic perspective, the validity of SPD was independent of the medical site of diagnosis.

Fifth, the second highest genetic risk score observed in our SPD sample was for ASD. We are unaware of prior reports of this association but it is consistent with the frequent overlap observed for schizotypal and autistic symptoms and signs (Barneveld et al., 2011). Of particular interest, FGRS_ASD_ was *significantly higher* in SPD cases than in schizophrenia, suggesting an etiologic relationship between the two syndromes. One way to assess the genetic relationship between SPD and ASD is to note that the level of FGRS_ASD_ in the SPD cases was 47% of that observed in ASD cases in this cohort from which we removed the SPD cases. This is considerably lower than the parallel figure of 72% for FGRS_SZ_ in cases of SPD v. schizophrenia. Our results suggest an important genetic relationship between SPD and ASD but not nearly as close as that seen for SPD and schizophrenia.

Sixth, the cases of SPD also had significant elevations of genetic risk for OCD, ADHD, and MD, which are consistent with prior reports of excess comorbidity of SPD with these disorders (Attademo & Bernardini, 2021; Bernardi et al., 2012; Fagel et al., 2013; Rosell et al., 2014). Compared to cases of schizophrenia, genetic risks were significantly elevated for ADHD and MD but not OCD.

Seventh, we evaluated, via LCA, whether our sample could be characterized by high levels of genetic heterogeneity such that most of our SPD cases had high genetic risk for only one of our four rarer disorders: SZ, ASD, OCD, and ADHD. Our best fit 3 class solution – defined as those at low, moderate, and high genetic risk -- was inconsistent with this hypothesis as the three classes differed quantitatively across all four genetic risks.

Finally, our follow-up data showed that 15% of our SPD cases developed schizophrenia, a rate moderately lower than the 20.9% reported over three treatment contacts in a Danish sample of SPD cases (Köhler-Forsberg et al., 2023) and considerably lower than the 33% seen over 20 years also in a Danish sample (Hjorthøj et al., 2018) of SPD cases. The literature also contains one ‘negative’ study as Zogbi et al., found that among those with an Attenuated Psychosis Syndrome, a diagnosis of SPD did not predict the development of schizophrenia (Zoghbi et al., 2019). Importantly, among the genetic predictors only the FRGS_SZ_ predicted conversion to SZ and, as seen in Fig. 2, the effect is relatively large. Consistent with epidemiological data showing a male excess in incident cases of schizophrenia (McGrath et al., 2004), we found males with SPD were over 50% more likely than females to progress to schizophrenia. A large effect was also found for the site of diagnosis. Since the site did not influence genetic risk in our SPD cases, it is likely that this effect was a result of the greater comorbidity in the SPD cases diagnosed in hospital and specialist *v.* primary care settings. Interestingly, in SPD cases, comorbidity with MD, ASD, and ADHD reduced the risk for the development of schizophrenia. Given prior evidence that a significant proportion of cases with drug-induced psychosis go on to develop schizophrenia especially if they have a high genetic risk for SZ (Kendler, Ohlsson, Sundquist, & Sundquist, 2019), we included drug use disorder (DUD) in cases of SPD as a possible predictor of the development of SZ. However, we did not find, as reported by Hjorthøj et al., that the SPD to schizophrenia conversion was significantly predicted by drug use disorder (Hjorthøj et al., 2018). However, their reported HR (1.34) is within the confidence intervals of our finding (HR = 1.19 (0.92; 1.53), so we cannot claim a definitive non-replication.

## Limitations

These results should be considered in the context of four methodological limitations. First, on the basis of the limited number of prior population prevalence estimates based on personal interviews (Sansone & Sansone, 2011; Torgersen et al., 2001), it is likely that SPD is substantially under-diagnosed in the Swedish healthcare system. This is perhaps unsurprising as SPD is not likely to be a diagnosis emphasized in the training of many physicians. We have a limited ability to determine the degree to which the diagnosed cases of SPD in Sweden are representative of all population cases. The fact that the conversion rate to schizophrenia is lower in our sample than seen in Danish registry cases of SPD suggests that our sample is not heavily biased toward severe cases and/or those with very schizophrenia-like presentations. Supportive of this conclusion is the high percentage of our SPD cases diagnosed by primary care physicians. We also show that our robust evidence of genetic risk for SZ in our SPD cases does not substantially result from a clinician bias to diagnose SPD in first-degree relatives of SZ probands. These findings provide some evidence to suggest that our SPD cases are not highly biased toward those with clinically severe schizophrenia-like presentations.

Second, our findings are also dependent on the quality of the non-SPD diagnoses from the Swedish registers which have generally been considered of high quality (Ludvigsson et al., 2011). The validity of SZ and OCD has been explicitly examined and supported (Ekholm et al., 2005; Lichtenstein et al., 2006; Rück et al., 2015). The validity of MD diagnoses is supported by its prevalence, sex ratio, familial correlations, and associations with psychosocial risk factors (Kendler, Ohlsson, Lichtenstein, Sundquist, and Sundquist, 2018; Sundquist, Ohlsson, Sundquist, and Kendler, 2017). However, we know of no studies assessing the validity of ADHD or ASD diagnoses in the Swedish medical registries although they have both been used in prior studies (e.g. (Daniels et al., 2008; Giacobini et al., 2023; Keil et al., 2010; Zhang-James et al., 2020)).

Third, the FGRS, a family phenotype-based measure to assess quantitative genetic risk distinct from PRS, has been now widely published (Kendler, Ohlsson, Sundquist, & Sundquist, 2021a; Kendler, Ohlsson, Sundquist, & Sundquist, 2021b; Kendler, Ohlsson, Sundquist, & Sundquist, 2023a; Kendler, Ohlsson, Bacanu et al., 2023b; Kendler, Ohlsson, Mościcki et al., 2023c; Kendler, Ohlsson, Sundquist, & Sundquist, 2023d; Kendler, Rosmalen, Ohlsson, Sundquist, & Sundquist, 2023e) with evidence that it is not highly sensitive to assumptions involved in its calculation, that the correction for cohabitation effects performs appropriately, and that the method agrees well with other similar statistical approaches (Hujoel, Gazal, Loh, Patterson, & Price, 2020; Krebs et al., 2023). Furthermore, recent empirical analyses and simulations demonstrate that the observed modest correlations between FRGS-like statistics and PRS from the iPsych study for psychiatric disorders are consistent with the hypothesis that current phenotype-based extended family measures, like those used in this study, and molecular-based polygene scores are both fallible measures of the same underlying large set of small effect genetic risk alleles (Krebs et al., 2023).

Above, we noted the frequent symptom overlap between schizotypal and autistic symptoms and signs (Cook, Zhang, & Constantino, 2020; Esterberg et al., 2008), but did not explicitly address the question of whether the high rates of FGRS_ASD_ in our SPD potentially reflects misdiagnosis, that is that some cases of SPD may really have ASD. To address this question, we excluded 13.8% of the SPD cases with comorbid ASD and calculated the mean FGRS_ASD_ for the remaining SPD cases. We found that it decreased only modestly and non-significantly from the value found for the full sample (0.304, 0.247–0.361) to 0.247 (0.188–0.306). This suggests that the elevated FGRS for ASD in our SPD cases is not likely a result of misdiagnosis or solely due to levels of SPD/ASD comorbidity.

## Conclusion

As diagnosed in the Swedish primary-care, specialist, and in-patient registries, SPD is a rare disorder. Affected individuals have high levels of comorbidity with ASD, OCD, ADHD, and MD and frequently demonstrate impaired educational attainment and subsequent psychosocial functioning. They have substantial elevations in their genetic risk for schizophrenia, independent of the specific registry in which they were diagnosed, but are also at increased genetic risk for ASD, MD, ADHD, and, to a lesser extent, OCD. Compared to cases of schizophrenia, they have significantly lower genetic risk of schizophrenia, but higher risks for ASD, ADHD, and MD. We could reject the hypothesis that our cases of SPD were genetically heterogeneous with most cases having an elevated genetic risk for only one of the four relatively rare disorders: SZ, ASD, ADHD, and OCD. Approximately 15% progress to schizophrenia over an 8-year follow-up. Predisposing factors include male sex, genetic risk for schizophrenia, and SPD diagnoses from an in-patient setting. Protective factors include comorbidity with ASD, MD, and ASD. Our results not only support the designation of SPD as a schizophrenia spectrum disorder but also suggest a potentially important etiologic link to ASD, and perhaps to ADHD, OCD, and MD.

## Supporting information

Kendler et al. supplementary material Kendler et al. supplementary material

## Supplementary material

The supplementary material for this article can be found at https://doi.org/10.1017/S0033291724000230.
