# Supplementary material for: The genetic epidemiology of schizotypal personality disorder
Source: Psychol Med. 2024 Feb 16;54(9):2144–51. doi: 10.1017/S0033291724000230 (PMC11413339; doi:10.1017/S0033291724000230)
Supplement: Kendler et al. supplementary material [file S0033291724000230sup001.docx]

Appendix

**Table 1**

**Description of Registers**

*National Patient Register*

In the 1960's the National Board of Health and Welfare started to collect information regarding in-patients at public hospitals, the National Patient Register (NPR). Initially it contained information about all patients treated in psychiatric care and approximately 16 percent of patients in somatic care. The register at that time covered six of the 26 county councils in Sweden. In 1984, the Ministry of Health and Welfare together with the Federation of County Councils decided a mandatory participation for all county councils. From 1987, NPR includes all in-patient care in Sweden. Since 2001, the register also covers outpatient doctor visits including day surgery and psychiatric care from both private and public caregivers. For more information, see https://www.socialstyrelsen.se/en/statistics-and-data/registers/register-information/the-national-patient-register/

*Primary Care Data*

We also used information from Primary Care. This is a research dataset including individual-level information on clinical diagnoses from primary health care centers. In the end of the follow-up period the registers covers almost 100% of the population. Figure 1 below show the percentage of the entire Swedish population that resides in counties with primary care data. For more information see: Sundquist, J., Ohlsson, H., Sundquist, K., Kendler, KS. Common adult psychiatric disorders in Swedish primary care where most mental health patients are treated. BMC Psychiatry 17, 235 (2017). https://doi.org/10.1186/s12888-017-1381-4

Figure 1

Percentage of the entire Swedish population that resides in counties with primary care data by Year

*
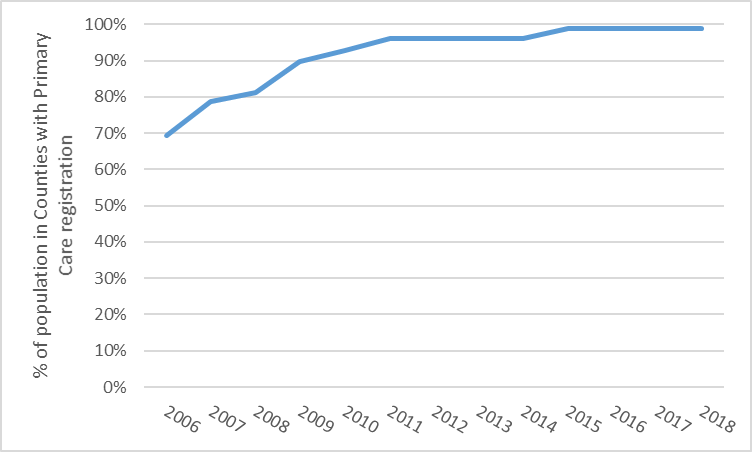
*

**Table 2 Definition of Variables**

|  | Registers Used | Definition |
| --- | --- | --- |
| Schizotypal Personality Disorder (SPD) | The Swedish Hospital Discharge Register (coverage 1997-2018); Outpatient Care Register (national coverage 2001-2018); Primary Care Registry (Partly coverage from 1999-2018) | ICD10: F21 |
| Schizophrenia (SZ) | The Swedish Hospital Discharge Register (coverage 1997-2018); Outpatient Care Register (national coverage 2001-2018); Primary Care Registry (Partly coverage from 1999-2018) | ICD-8: 295.1, 295.2, 2953, 295.9, 295.6; ICD-9: 295B, 295C, 295D, 295G, 295X; ICD-10: F200, F201, F202, F203, F205, F209 |
| Major Depression (MD) | The Swedish Hospital Discharge Register (coverage 1973-2018); Outpatient Care Register (national coverage 2001-2018); Primary Care Registry (Partly coverage from 1999-2018) | ICD-8: 296.2, 298.0, 300.4; ICD-9: 296.2, 296.4, 298.0, 300.4; ICD-10: F32, F33. |
| Obsessive-Compulsive Disorder [OCD] | The Swedish Hospital Discharge Register (coverage 1973-2017); Outpatient Care Register (national coverage 2001-2017); Primary Care Registry (Partly coverage from 1999-2017) | ICD-9: 300D; ICD-10: F42 |
| ADHD | The Swedish Hospital Discharge Register (coverage 1973-2017); Outpatient Care Register (national coverage 2001-2017); Primary Care Registry (Partly coverage from 1999-2017) | ICD-9: 314; ICD-10: F90 |
| Autism spectrum disorder (ASD) | The Swedish Hospital Discharge Register (coverage 1973-2017); Outpatient Care Register (national coverage 2001-2017); Primary Care Registry (Partly coverage from 1999-2017) | ICD-9: 299; ICD-10: F840, F841, F845, F849 |
| Drug Use Disorder (DUD)) | The Swedish Hospital Discharge Register (coverage 1973-2018); Outpatient Care Register (national coverage 2001-2018); Primary Care Registry (Partly coverage from 1999-2018); the Swedish Drug Register (2005-2018); the Swedish Mortality Register, and the Swedish Criminal Register (1973-2018) and the Swedish Suspicion Register (1998-2018) | Drug abuse (DA) was identified in the Swedish medical and mortality registries by ICD codes (ICD8: Drug dependence (304); ICD9: Drug psychoses (292) and Drug dependence (304); ICD10: Mental and behavioral disorders due to psychoactive substance use (F10-F19), except those due to alcohol (F10) or tobacco (F17)); in the Suspicion Register by codes 3070, 5010, 5011, and 5012, that reflect crimes related to DA; and in the Crime Register by references to laws covering narcotics (law 1968:64, paragraph 1, point 6) and drug-related driving offences (law 1951:649, paragraph 4, subsection 2 and paragraph 4A, subsection 2). DA was identified in individuals (excluding those suffering from cancer) in the Prescribed Drug Register who had retrieved (in average) more than four defined daily doses a day for 12 months from either of Hypnotics and Sedatives (Anatomical Therapeutic Chemical (ATC) Classification System N05C and N05BA) or Opioids (ATC: N02A). |
| Unemployment status | Longitudinal integrated database for health insurance and labour market studies (LISA) | In order to be entitled to unemployment benefits the following requirements needs to be fulfilled. The individual is: unemployed (fully or partially); able to work at least 3 hours a day and 17 hours a week; registered as a jobseeker at the Employment Service; prepared to take the offer of suitable work or employment; actively seeking for a suitable job.  In our models we consider an individual as unemployed if he/she has a registration anytime during the period 1992 and 2018 (during years they are above 20 years old) |
| Social welfare recipient | Longitudinal integrated database for health insurance and labour market studies (LISA) | Social assistance is defined as financial support under the Social Services Act. You can receive support for your upkeep and for other items that you need to have a reasonable standard of living. Examples of common situations when social assistance is given: As an income supplement to low-income families; for unemployed when other unemployment assistance is not provided or is insufficient; when sickness benefits are insufficient or not provided; to those who are bound by the children in the home and can not get childcare and therefore not can seek work. The variable is recorded at the family level, which means that all individuals in a family with social assistance will, in this report, be counted as recipients of Social welfare .  In our models we consider an individual as unemployed if he/she has a registration anytime during the period 1992 and 2018 |
| Divorce | Register of Total Population | Divorce is defined as the first divorce anytime in individuals who are registered as married |
| Not Married | Register of Total Population | Married is defined as the first marriage (could occur anytime during our follow up period) |
| Low Education | The longitudinal integration database for health insurance and labor market studies (LISA) from 1990-2017 and The Swedish Census from 1970. | Highest achieved education measured in 1-7 levels that in turn were translated into number of years of education and then standardized with mean 0 and SD 1 by gender and year of birth. In the registers the variable are as follows:  1-Pre-high school (7 years)  2-High School (9 years)  3-Upper Secondary School (11 years)  4-Upper Secondary School (12 years)  5-Post-secondary education (14 years)  6-Post-secondary education (17 years)  7- PhD education (21 years)  We consider low education as more than 1 SD below mean |
| Low Grades | The National School Registry. | Contains educational achievement (a grade point average) for all students at the end of basic high school (grade nine; usually at age 16). Students had an incentive to perform well in this school year because those with high grades were more likely to gain admission to the desirable upper secondary schools. For each year and by gender we standardized the grade score into a Z-score. From 1988 to 1997, the score was expressed on a scale between 1 (lowest) and 5 (overall mean was 3.2), and students were assessed by a peer referencing system. Grades awarded reflected the position of the student within Sweden and a set of correction factors were applied to ensure that the grades were equivalent between schools. Using this system, the grades had minimal grade inflation over time and were normally distributed. From 1998, the score was expressed on scale between 10 (lowest) and 320 (overall mean was 207) utilizing a criterion referenced system, in which students were assessed for their achievement of certain competencies. Scores were not standardized across schools or constructed to produce a normal distribution.  We consider low grades as more than 1 SD below mean |

**Table 3**

**Calculation of the Familial Genetic Risk Score (FGRS)**

|  |
| --- |
| The dataset for the calculations includes:  Column1 = Identification number of the proband (Born 1932-1995)  Column2 = Identification number of the relative (1st to 5th degree relatives)  Column3 = Proportion of shared additive genetic effects (0.03125 to 0.50) with the proband  Column4 = Year of Birth of relative  Column5 = Sex of relative  Column6 = Age at registration for trait  Column7 = Age at end of follow-up (2018-12-31 or age at death, or age at emigration whichever came first) |
| Step 1: Using all unique relatives with a registration for the disorder, we non-parametrically estimated the distribution of Age at first registration. The empirical distribution is used to obtain weights for relatives without a registration for the disorder, in order to account for the proportion of the time-at-risk period they had completed at the end of follow-up. For example, for relatives at age x at end of follow-up, the weight corresponds to the proportion of relatives registered for the trait that had been registration at age x. For relatives born prior to 1958 we subtracted age at the end of follow-up with the following formula: 1958 - Year of birth of relative. This modification was done in order to control for registration effects (i.e, most registers in Sweden start in 1973 suggesting that relatives from early birth cohorts do not have the possibility to be registered at younger ages). Note that all relatives with the disorder are weighted one. |
| Step 2: Transform the binary variable (trait yes/no) into a z-score based on the threshold for each trait. The underlying liability of the individual is not assessable. Instead we estimated the mean of the underlying liability to obtain sex and birth decade specific Z-scores for relatives with the trait registration and relatives without the trait. We generate n random numbers from a N(0, 1) distribution and estimate the mean for relatives registered with the disorder (i.e., mean of the observations above the threshold) and for relatives without a registration (i.e., mean of all observation below the threshold). The thresholds are calculated for each decade of birth and sex. |
| Step 3: Correct for cohabitation effects. To estimate the cohabitation effect (i.e. “shared environment”), we created a database with all individuals in the Swedish population born in Sweden 1955-1990. We also included the number of years, during ages 0-15, that individuals resided in the same household as their biological father. We thereby were able to define two kinds of families i) “not-lived-with” father families (offspring never resided for more than 1 year in the same household or in the same community as their biological father); ii) “lived-with” father (offspring resided a minimum of 13 year in the same household as their biological father. We performed a logistic regression model with the binary trait in offspring as outcome and the binary trait in father, type of father, and their interaction as predictors. We used the interaction term as the difference of effect between genes only and genes + environment. The same approach was performed for half-siblings where we compared those who were reared together versus reared apart. The following interaction terms were used in the calculations for each of our disorders:   \|  \| Parent/Children \| Siblings \| \| --- \| --- \| --- \| \| SZ \| 0.93 \| 0.84 \| \| MD \| 0.90 \| 0.89 \| \| OCD \| 0.79 \| 0.74 \| \| ADHD \| 0.42 \| 0.81 \| \| ASD \| 0.83 \| 0.61 \| |
| Step 4: Calculate the product for each relative using the four components:   1. Z-score (reflecting sex and year of birth adjusted rates) 2. Weight (reflecting the proportion of risk period they had completed) 3. Cohabitation effects 4. Proportion of shared genetic effects (0.03125 – 0.5) with the proband |
| Step 5: Average the product calculated in step 4 across all relatives to a proband |
| Step 6: Correct for the number of relatives. We multiplied the results from step 5 with a shrinkage factor. Shrinkage factor (SF): B/(B+A/C). It produces more shrinkage if B and C are small and A is large.   1. the variance of the z-score of the disorder across all relatives, 2. the variance in the mean z-score across all probands, 3. the weighted number of relatives for each proband (sum of Column 3 across each proband). |
| Step 7: Correct for difference by year of birth and county differences. There are 21 counties in Sweden. For each proband we used the county they had resided in during the maximum number of years (measured from 1969 and onwards) We standardized the risk score by year of birth and county of the proband into a z-score with mean 0 and SD 1. |

Table 4

K-means clustering for partition the FGRSs

We used a K-means clustering to partition the FGRSs into k clusters in which each individual belongs to the cluster with the nearest mean value. K-means clustering minimizes the within-cluster variances. We selected the number of clusters based on the elbow method (the inclusion of more clusters do not lead to a significant decrease in within-cluster sum of squares). For all four FGRSs the model resulted in 4 clusters. From the standardized score the thresholds for FGRSOCD was 0.67; 3.05; 7.22; for FGRSSZ 1.04, 4.16, 9.94; FGRSADHD 0.15, 1.55, 4.10 and ; FGRSASD 0.41, 2.30, 6.14.

Table 5 - Exact Values for Figure 1

| FGRS | SPD | SZ |
| --- | --- | --- |
| SZ | 0.419 (0.323; 0.516) | 0.583 (0.542; 0.624) |
| ASD | 0.304 (0.247; 0.361) | 0.202 (0.178; 0.227) |
| OCD | 0.119 (0.069; 0.169) | 0.095 (0.073; 0.116) |
| ADHD | 0.197 (0.154; 0.241) | 0.102 (0.083; 0.120) |
| MD | 0.291 (0.246; 0.335) | 0.145 (0.126; 0.164) |

Table 6

Fit of Latent Class Analysis

|  | 2 classes | 3 classes | 4 classes |
| --- | --- | --- | --- |
| AIC | 299.7 | 229.8 | 236.9 |
| BIC | 363.7 | 327.1 | 367.4 |
| Entropy | 0.61 | 0.65 | 0.63 |
|  |  |  |  |
